# The ArfGAP2/3 Glo3 and ergosterol collaborate in transport of a subset of cargoes

**DOI:** 10.1242/bio.011528

**Published:** 2015-05-11

**Authors:** Alejandro F. Estrada, Gopinath Muruganandam, Cristina Prescianotto-Baschong, Anne Spang

**Affiliations:** Growth & Development, Biozentrum, University of Basel, Klingelbergstrasse 70, 4056 Basel, Switzerland

**Keywords:** Sterol, Golgi, Endosomes, Small GTPases, Plasma membrane, Vesicle, Intracellular transport, Amino acid transporter, Lipid domains

## Abstract

Proteins reach the plasma membrane through the secretory pathway in which the trans Golgi network (TGN) acts as a sorting station. Transport from the TGN to the plasma membrane is maintained by a number of different pathways that act either directly or via the endosomal system. Here we show that a subset of cargoes depends on the ArfGAP2/3 Glo3 and ergosterol to maintain their proper localization at the plasma membrane. While interfering with neither ArfGAP2/3 activity nor ergosterol biosynthesis individually significantly altered plasma membrane localization of the tryptophan transporter Tat2, the general amino acid permease Gap1 and the v-SNARE Snc1, in a Δ*glo3* Δ*erg3* strain those proteins accumulated in internal endosomal structures. Export from the TGN to the plasma membrane and recycling from early endosomes appeared unaffected as the chitin synthase Chs3 that travels along these routes was localized normally. Our data indicate that a subset of proteins can reach the plasma membrane efficiently but after endocytosis becomes trapped in endosomal structures. Our study supports a role for ArfGAP2/3 in recycling from endosomes and in transport to the vacuole/lysosome.

## INTRODUCTION

Proteins expressed at the plasma membrane are synthesized into the endoplasmic reticulum (ER), transported to the Golgi and sorted into transport carriers to the plasma membrane. These transport carriers are either directly targeted to the plasma membrane or to endosomes. In the latter case, proteins are then routed to the plasma membrane through a different set of carriers, including recycling endosomes (Spang, 2015).

The small GTPase Arf1 is involved in most, if not all, vesicle generation events at the level of the Golgi apparatus. To perform its function Arf1 is activated by an Arf guanine nucleotide exchange factor (ArfGEF), which catalyzes the exchange of GDP by GTP on Arf1 and hence not only activate Arf1 but also stabilize its membrane association (Paris et al., 1997; Weiss et al., 1989). In the activated form, Arf1 recruits and interacts with its effector proteins, such as coat components, SNAREs and cargo proteins in order to drive vesicle formation. Arf1 activity is terminated by its interaction with a GTPase activating protein (ArfGAP), which stimulates the hydrolysis of GTP to GDP. Hence in a way only the complex of Arf1 with its GAP possesses significant GTPase activity (Spang et al., 2010).

Arf1 has multiple functions and it is thought that its temporal and spatial activation is mostly dependent on ArfGEFs. Yet, Arf1 also has numerous ArfGAPs, and in yeast the GAPs outnumber the GEFs by 2:1. Thus it is unlikely that the only function of the ArfGAPs is to turn off Arf1 activity. In fact, at least ArfGAP1 can time Arf1 inactivation through correlation to membrane curvature (Bigay et al., 2003). In addition, GAPs have been implicated in Arf1 recruitment to cargo, SNAREs and coat components; in case of the yeast ArfGAP2/3 Glo3 through the BoCCS region (Lanoix et al., 2001; Rein et al., 2002; Schindler et al., 2009). Thus ArfGAPs may be critical in determining the amplitude of Arf1 activity at its point of activation.

In *Saccharomyces cerevisiae* none of the ArfGAPs is essential for viability at standard laboratory growth conditions. ArfGAPs have overlapping functions and can substitute for each other (Poon et al., 1999; Poon et al., 2001). Concomitant loss of the ArfGAP1 homolog Gcs1 and the yeast ArfGAP2/3 Glo3 is lethal. Gcs1 and Glo3 have overlapping functions in retrograde transport from the Golgi to the ER, and can presumably also substitute each other, at least, at a subset of other intracellular localization (Poon et al., 1999). Yet, their mode of stimulation of Arf1 activity is not the same as the ArfGAP1 Gcs1 senses membrane curvature through ALPS motifs (Bigay et al., 2005), while the ArfGAP2/3 Glo3 interacts with coat components, cargo and SNAREs (Schindler et al., 2009). Moreover, they may also perform functions for which there is no substitute. For example, Δ*gcs1* and Δ*glo3* strains display growth defects at 15°C and Δ*gcs1* is defective in sporulation, while Δ*glo3* is respiratory defective (Connolly and Engebrecht, 2006; Ireland et al., 1994; Perrone et al., 2005; Poon et al., 1999).

We aimed to understand more about the regulation and specific function of the ArfGAP2/3 Glo3 by identifying its interaction partners. We found that Glo3 physically and genetically interacts with the C5 sterol desaturase Erg3. Ergosterol is the main sterol in the plasma membrane and can be envisaged as the yeast cholesterol. Ergosterol appears to be essential for the transport of a subset of plasma membrane localized proteins in Δ*glo3* cells. Our data indicate that ergosterol and Glo3 are required for proper recycling at endosomes and transport towards the vacuole/lysosome.

## RESULTS

### The ArfGAP Glo3 interacts with the C-5 sterol desaturase Erg3

**Fig. 1.**
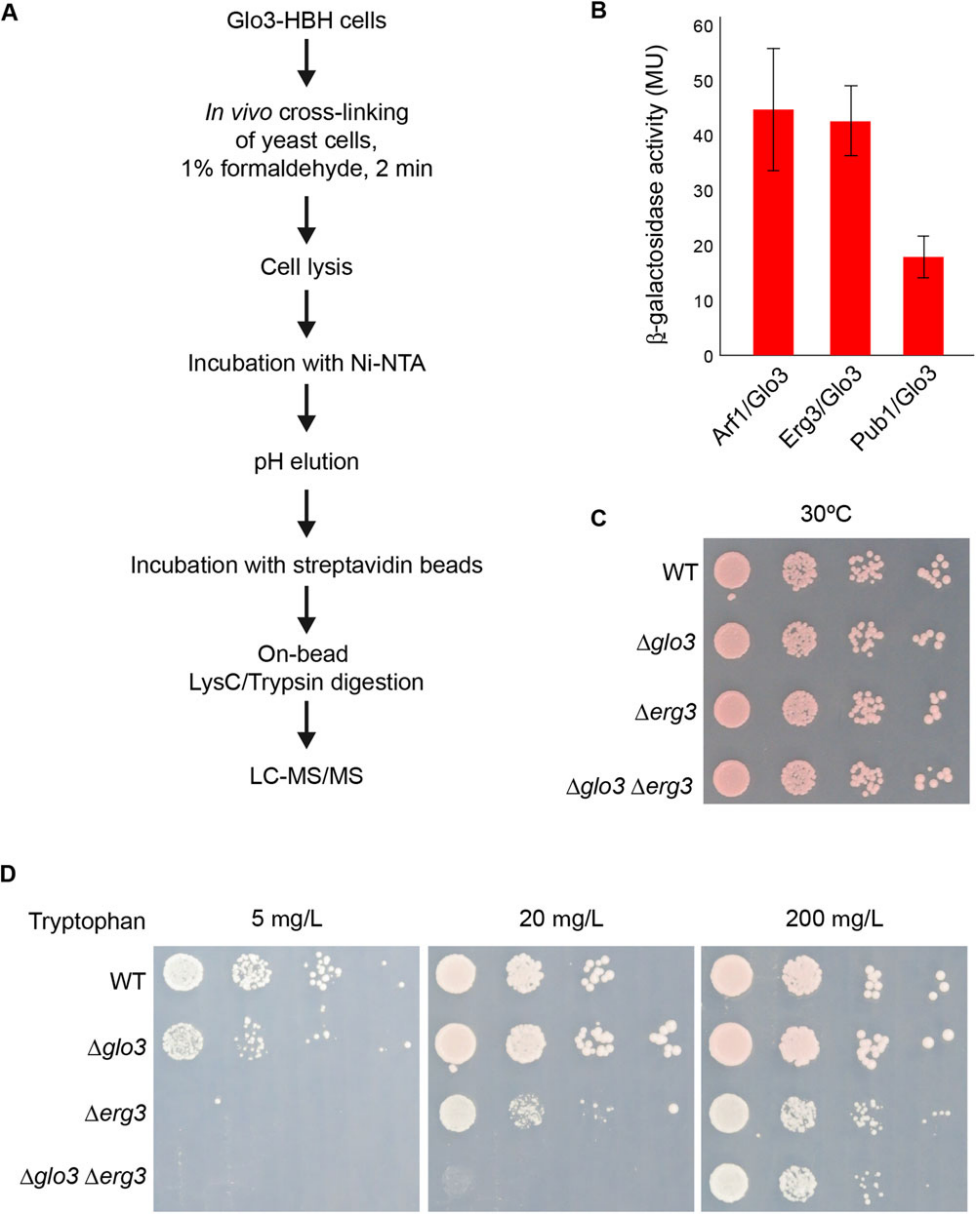
**The ArfGAP2/3 Glo3 interacts with the sterol C5 desaturase Erg3.** (A) Schematic outline of the HBH purification. (B) Erg3 and Glo3 interact in a yeast two-hybrid assay. β-galactosidase activity expressed as Miller units are given. Arf1 served as a positive and Pub1 as a negative control. Standard deviation of experiments performed in triplicates are given. (C) Growth of Δ*glo3*Δ*erg3* cells is not impaired on YPD. Drop test of indicated strains on YPD plates incubated at 30°C for 3 days. (D) *GLO3* and *ERG3* display synthetic genetic interaction on low tryptophan medium. Drop test of indicated strains on plates containing 5, 20 or 200 mg/l tryptophan; 200 mg/l is the standard tryptophan concentration in selective media.

To identify novel interactors of Glo3, we employed tandem-affinity purification after crosslinking with an HBH-tag followed by LC-MS/MS analysis (Fig. 1A) (Tagwerker et al., 2006). We have used this approach successfully before to identify a novel exomer-dependent cargo (Ritz et al., 2014) and regulators of processing body formation (Weidner et al., 2014). One of the proteins specifically enriched in the Glo3 fraction was the C-5 sterol desaturase Erg3. This protein plays an essential role in ergosterol biosynthesis, which is a major constituent of the plasma membrane and is the yeast counterpart of mammalian cholesterol (Nohturfft and Zhang, 2009). Since we detected the interaction between Glo3 and Erg3 by crosslinking, we verified the observation by a yeast two-hybrid analysis. Erg3 interacted with Glo3 to a similar level as the positive control Arf1, while the β-galactosidase activity was much lower with Pub1, which served a negative control (Fig. 1B). Thus, Glo3 potentially interacts with Erg3 also *in vivo*.

To extend our results, we tested for genetic interactions between *ERG3* and *GLO3* and generated a Δ*glo3* Δ*erg3* double mutant. The growth of this strain was not more impaired on rich medium (YPD) than of the individual Δ*glo3* and Δ*erg3* deletion strains (Fig. 1C). Ergosterol has been shown to be important for cell surface expression of the tryptophan transporter Tat2, which is essential for tryptophan uptake in yeast (Daicho et al., 2009). As observed previously for another mutant in ergosterol synthesis, Δ*erg2* (Daicho et al., 2009), Δ*erg3* cells were unable to grow in the presence of low tryptophan concentrations (Fig. 1D). This phenotype was enhanced in the Δ*glo3* Δ*erg3* double deletion strain, which was unable to grow at intermediate tryptophan levels, indicating that *GLO3* and *ERG3* interact genetically. Since Glo3 and Gcs1 have partially overlapping functions (Poon et al., 1999), we tested next whether *GCS1* would interact genetically with *ERG3*. This genetic interaction was, in fact, much stronger as the Δ*gcs1*Δ*erg3* double deletion was lethal (data not shown). Our data indicate a connection between the ergosterol synthesis and ArfGAPs.

### Tat2-GFP is mislocalized in Δ*glo3* Δ*erg3* cells

**Fig. 2.**
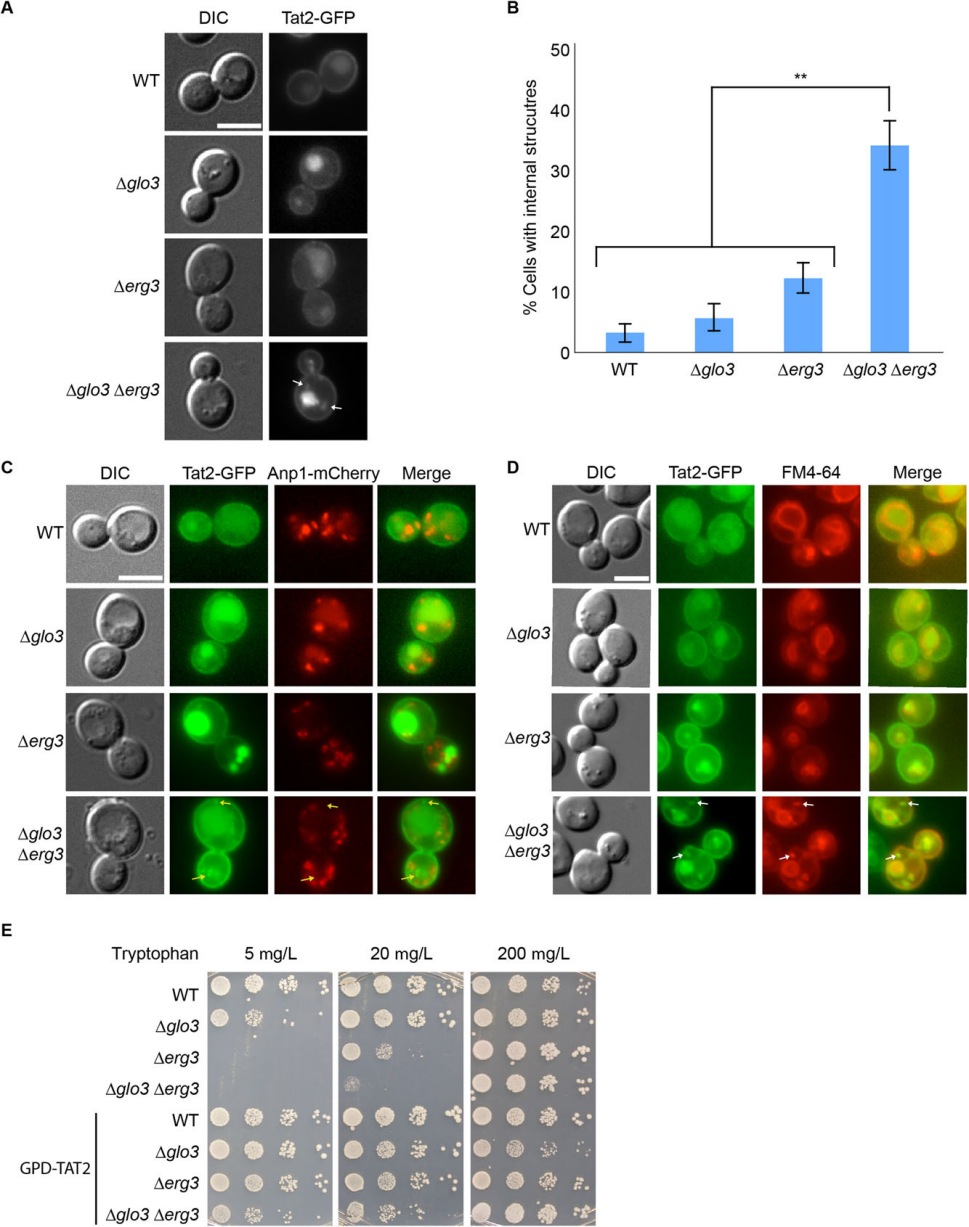
**The localization of the tryptophan permease Tat2 is impaired in Δ*glo3*Δ*erg3* cells.** (A) Tat2 accumulates in intracellular foci in Δ*glo3*Δ*erg3* cells. The localization of Tat2-GFP was assessed in early- to mid-log phase growing cells of different strains. (B) Quantification of A. The data of at least three independent experiments in which≥100 cells were counted per strain are displayed. Error bars represent standard deviation. The p-value corresponds to<0.01. (C) Tat2 does not accumulate in the Golgi. Double labeling of Tat2-GFP and the Golgi marker Anp1-mCherry. Arrows point to non-overlapping signals. (D) Tat2 accumulates in endocytic compartments. Double staining of Tat2-GFP and the lipophilic dye FM4-64, marking endocytic compartments. Arrows point to overlapping signals. (E) Overexpression of Tat2 rescues the growth defect of Δ*erg3* and Δ*glo3*Δ*erg3* cells on low tryptophan plates. Drop assay of indicated yeast strains on selective media containing different concentration of tryptophan; 200 mg/l being the standard concentration. The scale bars in A, C and D represent 5 µm.

An obvious explanation for the sensitivity to low tryptophan levels is that the permeaseTat2 may not reach the plasma membrane efficiently and hence not enough tryptophan would be taken up into the cell. To test this hypothesis, we analyzed strains in which Tat2 was chromosomally appended with GFP. Tat2-GFP was present at the plasma membrane and in the vacuole in wild-type cells (Fig. 2A). In both Δ*erg3* and Δ*glo3* cells the equilibrium of the steady-state localization shifted towards the vacuole. In contrast, small bright structures distinct from the vacuole were observed in the double mutant (Fig. 2A,B). These structures did not co-localize with the Golgi marker Anp1-mCherry (Fig. 2C), indicating that Tat2 transport through the Golgi remains unaffected by the lack of ergosterol and Glo3. Since a considerable portion of Tat2 appeared still at the plasma membrane, we tested whether the Tat2 accumulation occurred in endosomes. We used the lipophilic dye FM4-64 to mark the endocytic pathway. The bright internal Tat2 dots co-localized with FM4-64 in Δ*glo3* Δ*erg3* cells (Fig. 2D), indicating that transport from the plasma membrane to the vacuole might be delayed in the double compared to each single mutant. Moreover, these data suggest that Tat2 may not be fully functional in the absence of ergosterol and hence endocytosed more rapidly. If this assumption was correct, increasing Tat2 levels should alleviate the growth phenotype of Δ*erg3* and Δ*glo3*Δ*erg3* on low TRP plates. Overexpression of Tat2 rescued growth defect of Δ*glo3* Δ*erg3* (Fig. 2E). Our data so far indicate that loss of Glo3 aggravates the Δ*erg3* phenotype in terms of Tat2 localization and suggests a function of Glo3 at endosomes.

### TORC1 is signaling is affected in Δ*erg3* cells

Starvation induces the degradation of high affinity amino acid permeases such as Tat2 (Schmidt et al., 1998). This degradation is prevented by Tat2 phosphorylation through the TORC1-activated kinase Npr1 under normal growth conditions (Schmidt et al., 1998). Since the steady state GFP signal was more prominent in the vacuole in Δ*erg3* cells and hence Tat2 may be less stable, we wondered whether TORC1 signaling would be affected under those conditions. Deletion of *ERG3* caused the cells to be sensitive to the TORC1 inhibitor, rapamycin (supplementary material Fig. S1A). Surprisingly, additional loss of *GLO3* slightly alleviated the Δ*erg3* rapamycin-sensitivity.

To assess TORC1 activity on a shorter time scale, we determined the phosphorylation status of the direct target of the complex, Sch9 (Urban et al., 2007). Sch9 is phosphorylated in response of TORC1 activation and these changes in TORC1-dependent phosphorylation can be detected by immunoblot (Stracka et al., 2014). The non-phosphorylated form of Sch9 accumulated faster in Δ*erg3* (supplementary material Fig. S1B). Thus TORC1 signaling is reduced in Δ*erg3* mutant cells. However, this faster dephosphorylation was not reverted in Δ*glo3* Δ*erg3* cells indicating that Glo3 may not affect TORC1 activity directly.

### Gap1 requires Glo3 and ergosterol for efficient plasma membrane localization

**Fig. 3.**
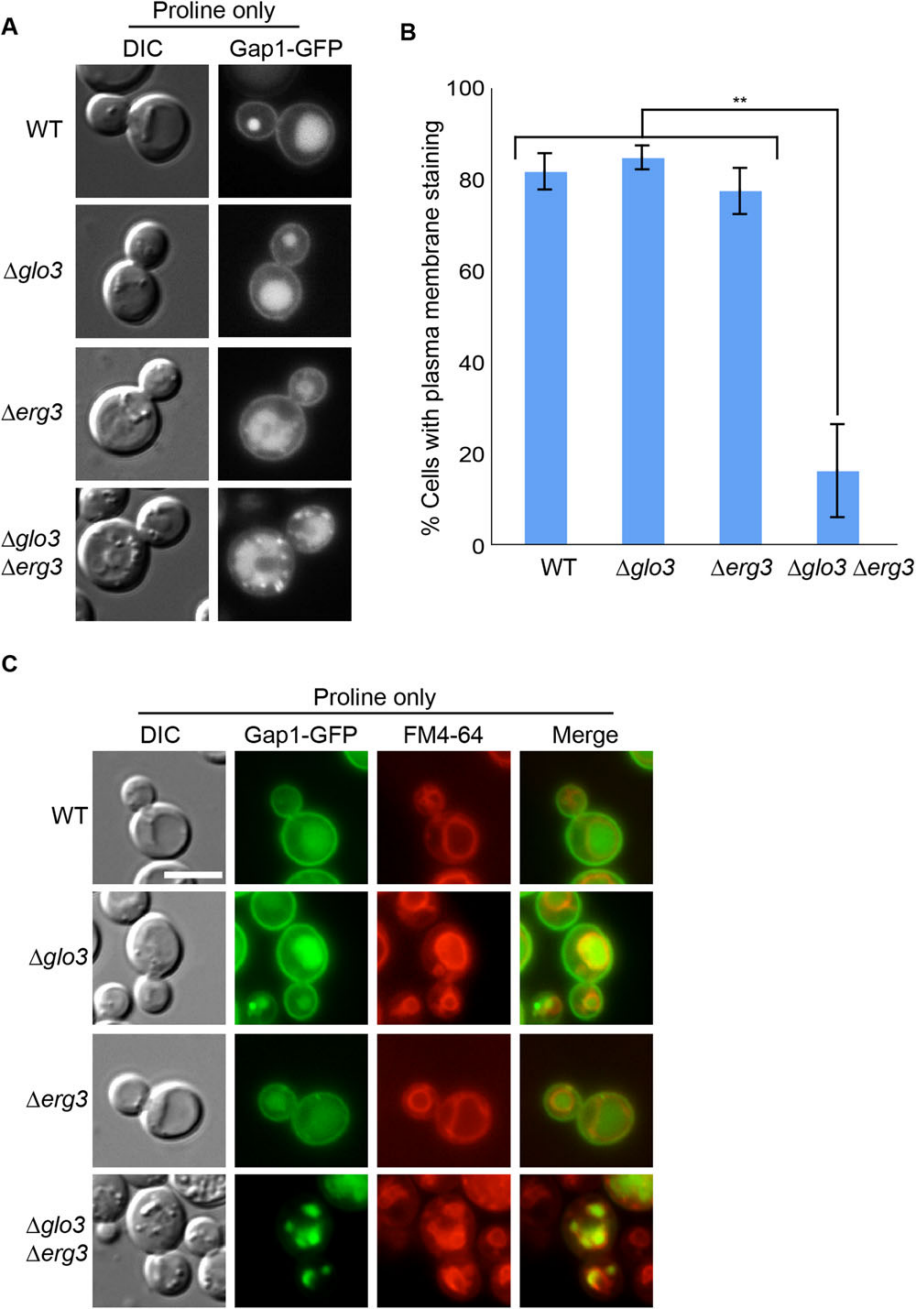
**The localization of the general amino acid permease Gap1 is impaired in Δ*glo3*Δ*erg3* cells.** (A) Gap1 accumulates in intracellular foci in Δ*glo3*Δ*erg3* cells. The localization of Gap1-GFP was assessed in early- to mid-log phase growing cells of different strains in selective media, which only contained proline as nitrogen source. (B) Quantification of A. At least 100 cells in each of three independent experiments were counted. Error bars represent standard deviation. The p-value corresponds to<0.01. (C) Gap1 accumulates in endocytic compartments. Double staining of Gap1-GFP and the lipophilic dye FM4-64, marking endocytic compartments. Scale bar represents 5 µm.

Next we wondered whether the Δ*glo3* Δ*erg3* deletion specifically affects Tat2 localization or has a more general effect on the transport of proteins. First, we decided to determine the localization of another amino acid permease, Gap1. The general amino acid permease Gap1 is degraded in the vacuole under rich nutrient conditions but is expressed at the plasma membrane under nutrient limiting conditions such as in the presence of proline as the sole nitrogen source (De Craene et al., 2001) (Fig. 3A). Deletion of *GLO3* or *ERG3* did not interfere with Gap1 plasma membrane localization, while less Gap1 was present at the plasma membrane in the double mutant (Fig. 3A,B). In particular small bright foci were present in the Δ*glo3* Δ*erg3* cells. Again these foci corresponded to endosomal structures because they were positive for FM4-64 (Fig. 3C). Thus, similar to Tat2, Gap1 plasma membrane localization is dependent on the presence of ergosterol and the ArfGAP2/3 Glo3.

### Not all plasma membrane proteins depend on ergosterol and Glo3 for proper localization

**Fig. 4.**
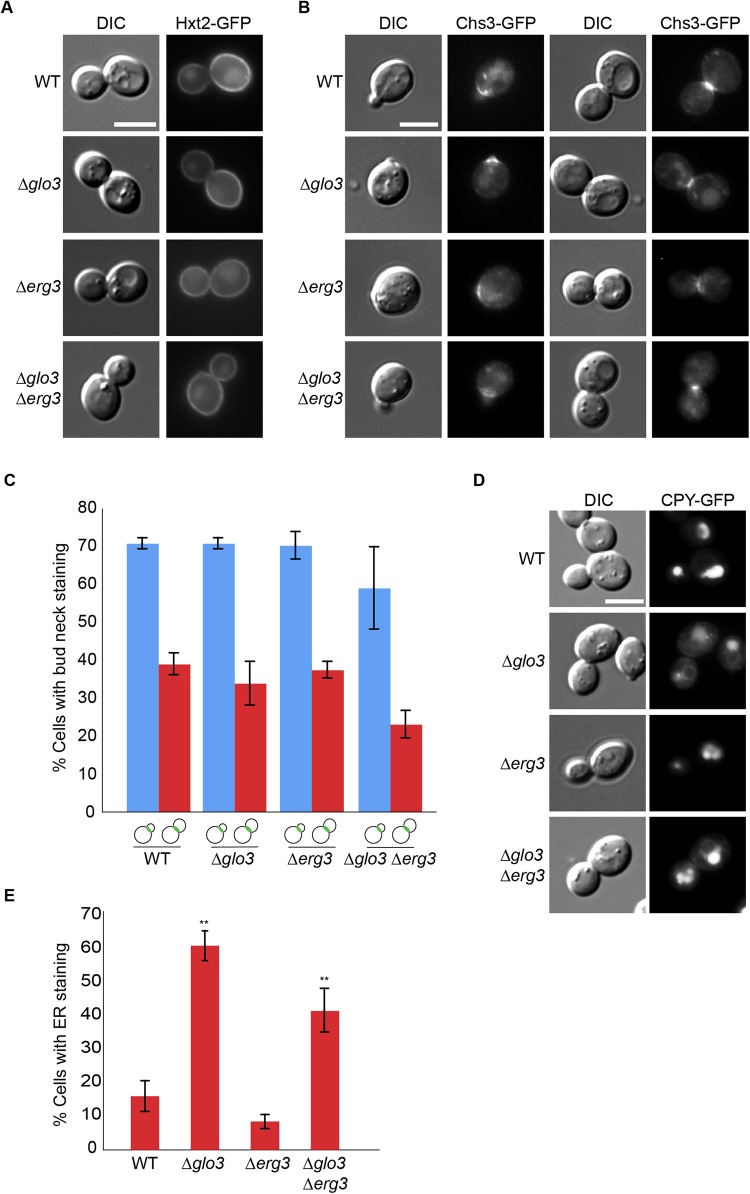
**Not all cargo depends on Glo3 and ergosterol for proper plasma membrane localization.** (A) The localization of glucose transporter Hxt2 is not impaired in Δ*glo3*Δ*erg3* cells. Hxt2 was appended chromosomally with GFP. Early- to mid-log phase grown cells were analyzed by microscopy. (B) Chitin synthase III transport to the plasma membrane and recycling through the TGN is not perturbed in Δ*glo3*Δ*erg3* cells. Early- to mid-log phase grown cells expressing Chs3-GFP were analyzed by microscopy. Chs3 is localized to the bud neck in small and in large budded cells. (C) Quantification of Chs3 bud neck localization in different strains. The data of at least three independent experiments in which≥100 cells per cell-cycle stage were counted are presented. Error bars represent standard deviation. Small and large budded cells are schematically represented. (D) Carboxypeptidase Y (CPY) transport is not aggravated in Δ*glo3*Δ*erg3* cells. CPY-GFP transport to the vacuole was assessed in indicated strains. In all cases vacuolar localization was observed, albeit with a varying degree of efficiency. CPY accumulated in the ER in Δ*glo3* and Δ*glo3*Δ*erg3* cells. (E) Quantification of the phenotype displayed in D. At least 100 cells in each of three independent experiments were counted. Error bars represent standard deviation. The p-value corresponds to<0.01. The scale bars in A, B and D represent 5 µm.

Next, we examined the localization of the hexose transporter, Hxt2. As previously observed, Hxt2-GFP is localized at the plasma membrane and to a lesser extent in the vacuole (Zanolari et al., 2011). Hxt2 was present at the plasma membrane in all strains tested (Fig. 4A), indicating that ergosterol is not generally necessary to transport or keep proteins at the plasma membrane.

Because Hxt2 does not cycle between internal compartments but after endocytosis is degraded in the vacuole, we tested a plasma membrane protein, which cycles between internal compartments and the plasma membrane. Such a cargo is the chitin synthase Chs3, which cycles between the bud neck at the plasma membrane and the TGN in a cell-cycle dependent manner, and recycles constantly through the endosomal system (Valdivia et al., 2002; Zanolari et al., 2011). Chs3 appeared to be somewhat less efficiently exported from the ER in Δ*erg3* cells as we could observe a weak Chs3-GFP signal in the ER, but otherwise Chs3 localization was unaffected by either single mutant (Fig. 4B,C). In the Δ*glo3* Δ*erg3* double mutant we observed a rather small drop in bud neck localization of Chs3 (Fig. 4B,C), indicating that loss of *GLO3* and *ERG3* does not severely affect Chs3 localization and its retrieval to the TGN.

Given that Tat2 and Gap1 were detected in endosomal compartments en route to the vacuole in Δ*glo3* Δ*erg3* cells, we asked whether transport of the vacuolar carboxypeptidase Y (CPY) that reaches its destination via the TGN and endosomes (Bryant et al., 1998) is altered. Using pulse chase analysis it has been shown previously that CPY transport to the vacuole is delayed in Δ*glo3* cells (Poon et al., 1999). While CPY was partially retained in the ER in Δ*glo3* and Δ*glo3*Δ*erg3* cells, no accumulation in endosomes was detected in either strain (Fig. 4D,E). Thus, we conclude that Glo3 and ergosterol cooperate only on the localization of a subset of membrane proteins.

### Pma1 can accumulate in the ER in Δ*glo3* Δ*erg3* cells

**Fig. 5.**
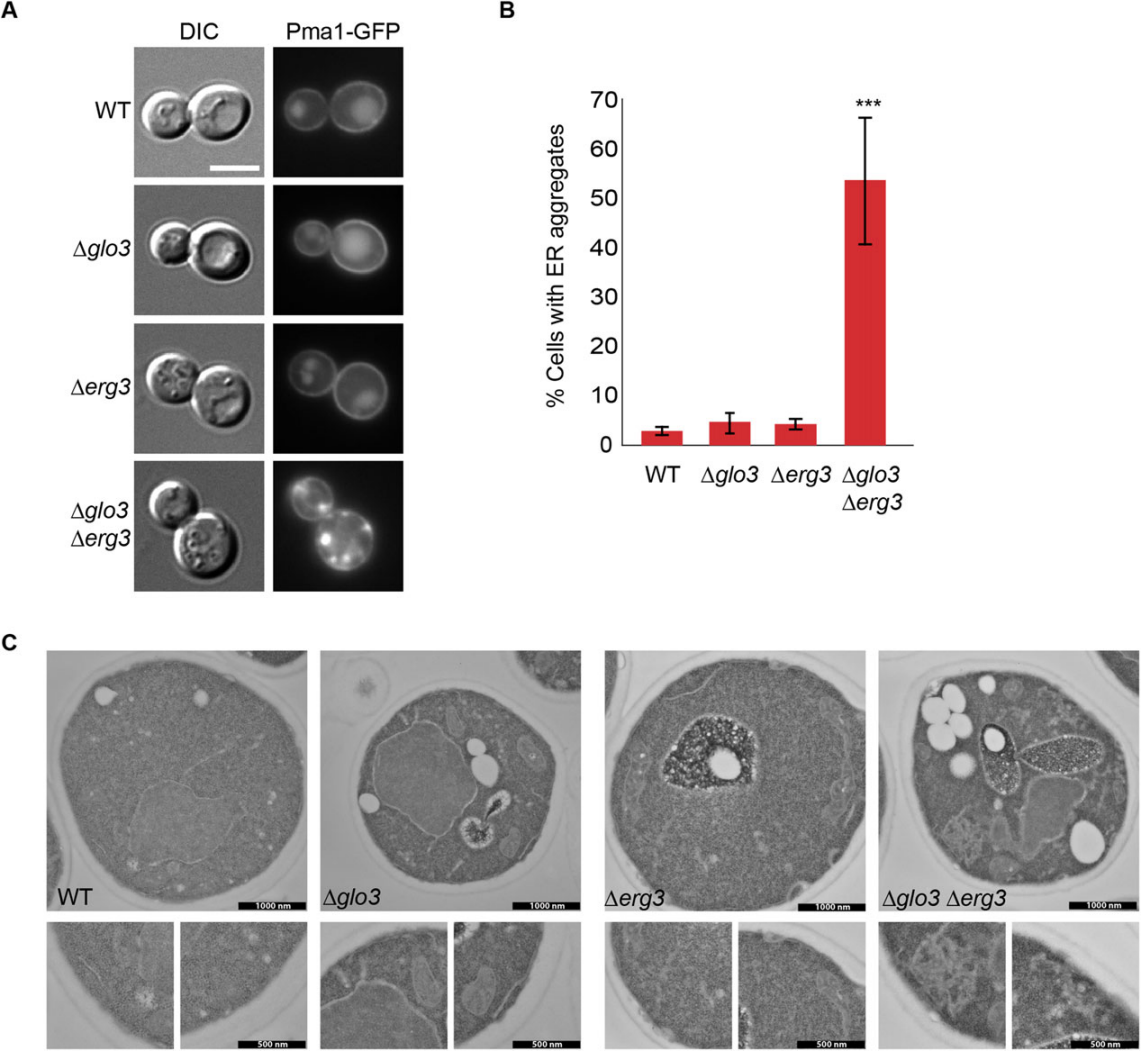
**In Δ*glo3*Δ*erg3* cells, Pma1 accumulates in distinct areas in the ER.** (A) Pma1-GFP is retained internally in Δ*glo3*Δ*erg3* cells. Early- to mid-log phase grown cells were analyzed by microscopy. Scale bar represents 5 µm. (B) Quantification of the phenotype displayed in A. At least 100 cells in each of three independent experiments were counted. Error bars represent standard deviation. The p-value corresponds to<0.001. (C) Pma1 causes proliferation of ER subdomains. Electron microscopy analysis of strains expressing Pma1-GFP. The scale bar in the low magnification is 1 µm and for the enlargements 500 nm.

**Fig. 6.**
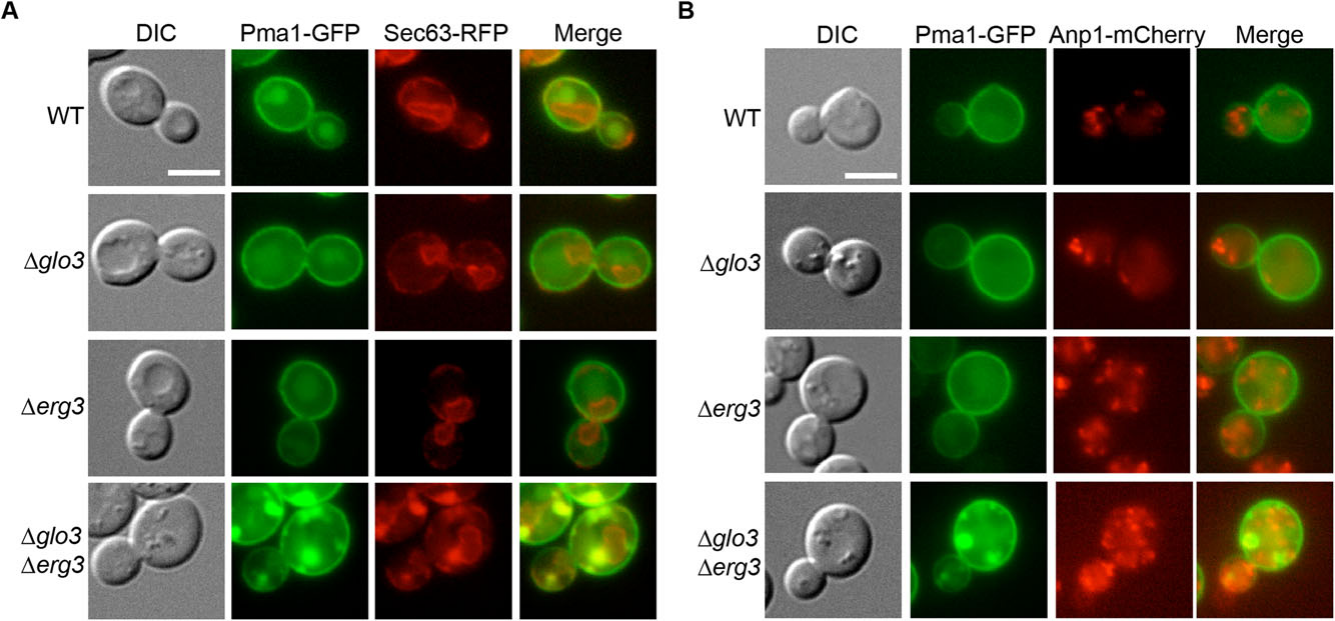
**Pma1-GFP does accumulate in a compartment enriched for an ER marker.** (A) Pma1-GFP is retained in ER subcompartments in Δ*glo3*Δ*erg3* cells. Cells co-expressing Pma1-GFP and the ER marker Sec63-RFP were analyzed. (B) The Pma1 accumulations in Δ*glo3*Δ*erg3* cells are not positive for the Golgi marker Anp1. Early- to mid-log phase grown cells expressing Pma1-GFP and Anp1-mCherry were analyzed by microscopy. Scale bars represents 5 µm.

Except for Tat2 and Gap1, the cargoes that we analyzed so far do not depend on the presence of ergosterol. Thus it is conceivable that another protein that would be localized in a lipid microdomain could show a similar mislocalization than amino acid permeases in Δ*glo3* Δ*erg3*. The plasma membrane ATPase Pma1 was shown to be transported to the plasma membrane in ergosterol- and sphingolipid- rich secretory vesicles (Surma et al., 2011). Yet, interfering with ergosterol synthesis did not cause a decrease in plasma membrane localization of Pma1 (Gaigg et al., 2005). Consistent with this previous report, Pma1 reached the plasma membrane in a Δ*erg3* strain indistinguishable form wild type cells (Fig. 5A). However, in Δ*glo3* Δ*erg3* cells, we observed Pma1 aggregates, especially in cells that seemed to express high levels Pma1-GFP. This phenotype was not observed in wild type or Δ*glo3* cells (Fig. 5A,B). To further characterize this aggregates, we analyzed the strains by electron microscopy. Both Δ*glo3* and Δ*erg3* mutants have a slight ER morphology defect at the ultrastructural level (Fig. 5C). In the Δ*glo3* Δ*erg3* double mutant however, a strong accumulation of ER membranes that were organized in tubules was observed (Fig. 5C). We infer that these membrane accumulations correspond to the Pma1 aggregations observed by light microscopy. To confirm this notion, we labeled the ER with Sec63-RFP and found that Pma1-GFP co-localized with the ER marker (Fig. 6A). At steady state, Glo3 is predominantly localized at the Golgi with a significant cytoplasmic pool (Fig. S2) (Huh et al., 2003). Therefore we tested whether these membrane accumulations would also contain Golgi membranes; however, we unable to detect Anp1 in the Pma1 aggregates (Fig. 6B). Therefore, we conclude that Pma1 can aggregate in the ER in the absence Glo3 and Erg3. This effect is not due to the GFP tag on Pma1 because expression of Pma1 without the tag was sufficient to drive the ER membrane accumulations (supplementary material Fig. S3). Our data suggest that loss of Glo3 and Erg3 sensitizes intracellular trafficking pathways in particular for proteins that rely on ergosterol-containing membrane domains.

### Plasma membrane localization of the SNARE Snc1 is reduced in Δ*glo3* Δ*erg3* cells

**Fig. 7.**
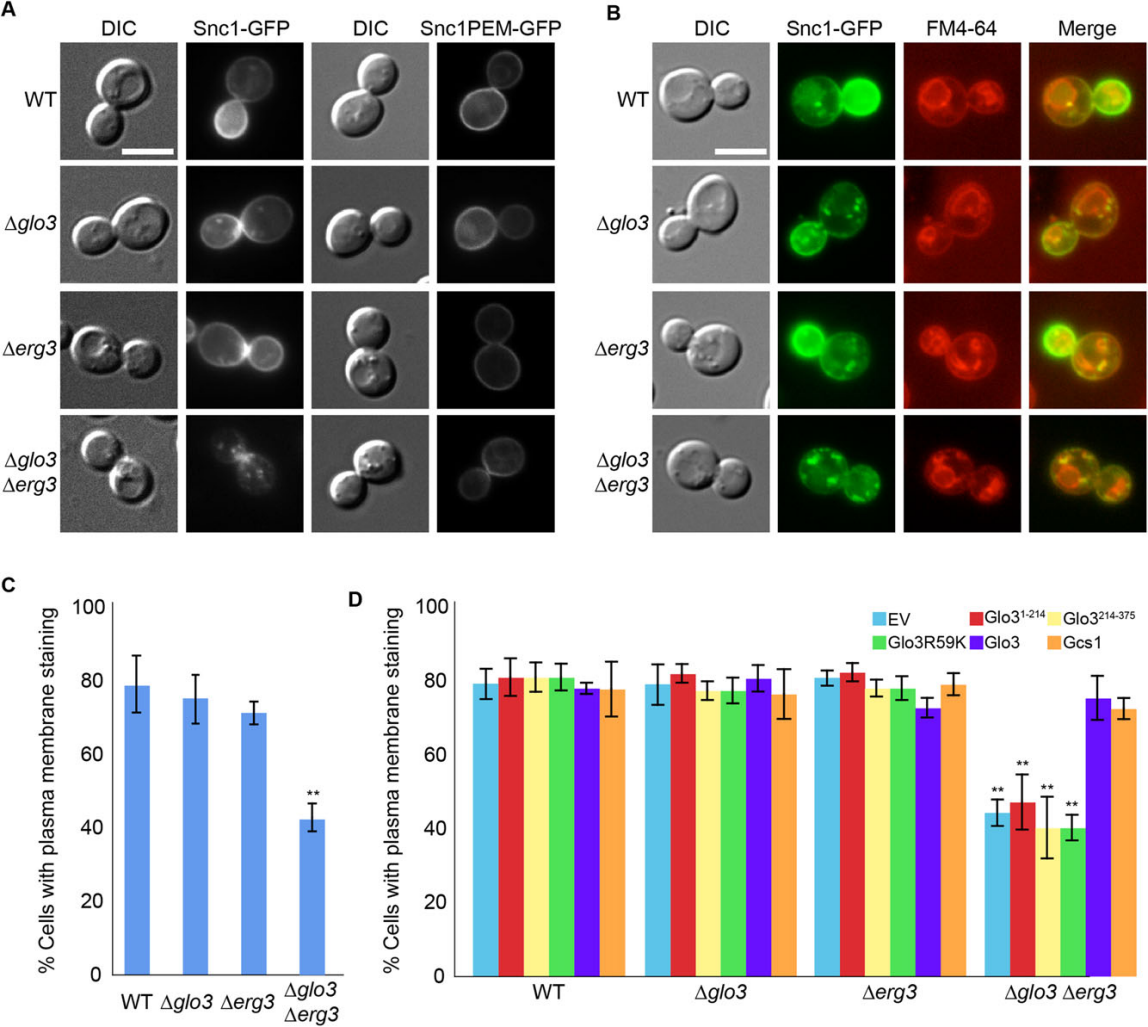
**Snc1 recycling to the plasma membrane requires Glo3 and ergosterol**. (A) Snc1 recycling is impaired in Δ*glo3*Δ*erg3* cells. Snc1-GFP or the endocytosis deficient Snc1PEM-GFP were analyzed in logarithmically growing cells. (B) Snc1-GFP accumulates in endosomes. Co-labeling of Snc1-GFP and FM4-64, which marks endocytic compartments. (C) Quantification of Snc1-GFP plasma membrane localization. (D) The BoCCS region and the GAP domain are both required for proper Snc1 plasma membrane localization. Δ*glo3*Δ*erg3* Snc1-GFP cells were transformed with an empty plasmid or plasmids expressing either wild-type Glo3 or mutant version of Glo3. Plasma membrane localization of Snc1-GFP was quantified. (C,D) At least 100 cells in each of three independent experiments were counted. Error bars represent standard deviation. The p-value corresponds to<0.01. The scale bars in A and B represent 5 µm.

Tat2 and Gap1 were present in endosomal structures in Δ*glo3*Δ*erg3* cells. One reason for the phenotype could be that recycling back to the plasma membrane could be impaired under these conditions. To test this hypothesis we analyzed the localization of the v-SNARE Snc1, which is required for fusion of transport vesicles with the plasma membrane (Protopopov et al., 1993). Again neither single mutant showed a defect in Snc1 localization (Fig. 7A,B). In contrast the Δ*glo3*Δ*erg3* double mutant retained most of the Snc1 in internal compartments that were accessible for FM4-64 (Fig. 7A–C). To exclude that Snc1 may not reach the plasma membrane in Δ*glo3* Δ*erg3* cells, we determined the localization of a Snc1mutant that cannot be endocytosed, Snc1PEM (Lewis et al., 2000). Snc1PEM reached the plasma membrane efficiently in all strains tested (Fig. 7A). These data are consistent with the results on Hxt2 and Chs3 that also were correctly localized in Δ*glo3* Δ*gcs1* cells and indicate that exocytosis per se is not majorly affected under those conditions. Our data suggest a shift in the dynamic equilibrium of Snc1 localization that could be either brought about by a delay in recycling from endosomes to the TGN or a more rapid endocytosis, or a combination of both.

### The ArfGAP activity of Glo3 is necessary but not sufficient maintain Snc1 plasma membrane localization

Glo3 contains two regions that are essential for its function. First the GAP domain, which stimulates the GTP hydrolysis on Arf1 and the BoCCS region, which provides the interaction surface for cargo, coatomer and SNARE proteins (Poon et al., 1999; Schindler et al., 2009). We asked whether the GAP activity required for plasma membrane localization of Snc1. When we expressed the GAP-dead mutant Glo3R59K (Lewis et al., 2004) or the BoCCS region (Glo3^214–375^) in Δ*glo3* Δ*erg3*, Snc1 remained in internal structures, indicating that the GAP activity was required for efficient Snc1 transport (Fig. 7D). However, expression of the GAP domain (Glo3^1–214^) was not sufficient to rescue the Δ*glo3* Δ*erg3* phenotype.

Glo3 and Gcs1 have overlapping functions (Poon et al., 1999). Moreover Snc1 is confined to internal structures in a Δ*gcs1* (Robinson et al., 2006). Therefore, we tested whether overexpression of *GCS1* would suppress the Δ*glo3* Δ*erg3* phenotype. Snc1 plasma membrane localization was rescued by increased levels of Gcs1. Taken together our data suggest role of ArfGAP2/3 at endosomes in the transport of a subset of cargoes, whose plasma membrane residence time might be sensitive to ergosterol levels.

## DISCUSSION

The ArfGAP2/3 Glo3 has a well-established role in retrograde transport form the Golgi to the ER and is present of COPI-coated vesicles (Lewis et al., 2004; Poon et al., 1999). This role is also conserved in mammalian cells (Frigerio et al., 2007; Kliouchnikov et al., 2009; Weimer et al., 2008). We have uncovered a requirement for Glo3 on endosomes. This requirement was only revealed in a background in which ergosterol synthesis was defective. In a Δ*glo3*Δ*erg3* strain, the plasma membrane localization of a subset of cargoes such as Tat2 and Gap1 and the v-SNARE Snc1 were impaired as they accumulated in endosomes. Exocytosis of at least Snc1 did not seem to be affected under these conditions because the endocytosis-defective Snc1 mutant, Snc1-PEM reached the plasma membrane efficiently. To our knowledge this is the first link between an ArfGAP and sterols.

The role of ergosterol in transport of the tryptophan permease Tat2 has been reported before, as cells deficient for *ERG2* or *ERG6* were unable to grow on medium with low tryptophan levels (Daicho et al., 2009; Umebayashi and Nakano, 2003). Under these conditions, Tat2 reached the early endosome and was then missorted into late endosomes/multivesicular bodies. Erg3 is downstream of Erg6 and Erg2 in the ergosterol synthesis pathway. It is conceivable that Tat2 still reaches the plasma membrane in Δ*erg3* cells because episterol, the substrate of Erg3, may already partially fulfill ergosterol function in the membrane. However Tat2 may only be partially functional, if at all, because Δ*erg3* cells still fail to grow on low tryptophan plates, and overexpression of *TAT2* rescued this phenotype.

Yet not the transport of all cargoes is affected in Δ*glo3*Δ*erg3* cells: The localization of the chitin synthase Chs3 and the glucose transporter Hxt2 was independent of Glo3 and Erg3. Chs3 localizes at incipient bud site and the bud neck in G1 and at the end of M phase (Chuang and Schekman, 1996; Reyes et al., 2007; Trautwein et al., 2006; Zanolari et al., 2011). This cell-cycle regulated localization is dependent on constant endocytosis and recycling through the TGN (Valdivia et al., 2002). Since Chs3 localization was not altered in Δ*glo3*Δ*erg3* cells, retrograde transport from endosomes to the TGN may not be generally perturbed. However, the v-SNARE Snc1, which also needs to recycle from endosomes to the Golgi, was retained in endocytic structures in Δ*glo3*Δ*erg3* cells. It is conceivable that Chs3 and Snc1 use different routes back to the TGN. This notion is supported by the finding that Snc1 was retained in endosomes while Chs3 transport was functional in cells in which the ArfGAP1 *GCS1* was deleted (Robinson et al., 2006). Thus, Δ*glo3* Δ*erg3* cells display the same phenotype as Δ*gcs1* cells in terms of Snc1 localization. One possible explanation is that Glo3 and ergosterol are required in the same recycling pathway to the Golgi than Gcs1, but Gcs1 has a more prominent role. In accordance with this hypothesis, double deletions of Δ*gcs1* with either Δ*glo3* or Δ*erg3* are lethal. Glo3 was recently implicated retrograde transport from late endosomes to the TGN (Kawada et al., 2015). Moreover ArfGAP3, the mammalian homolog of Glo3 was associated with the recycling of the cation independent mannose-6-phosphate receptor (CIMPR) (Shiba et al., 2013). Glo3 could be required for the recruitment of Snc1 into transport vesicles as Glo3 can induce a conformational change on Snc1 to promote Arf1 binding *in vitro* (Schindler and Spang, 2007). In a variation of this model, Erg3 and Glo3 would act in a parallel recycling pathway to Gcs1. For example, Gcs1 could function in recycling from early and Glo3/ergosterol from late endosomes.

An alternative scenario is that the permeases and Snc1 are more rapidly endocytosed in the absence of ergosterol. Direct recycling to the plasma membrane or through the TGN would still be at least partially functional and hence no strong defect would be detectable. When Glo3 is missing under these conditions, endosomal sorting may be delayed causing the accumulation of cargoes in endocytic compartments. The endosomes were mostly in close proximity to the yeast lysosome, the vacuole, indicating transport to the vacuole might be slowed down. This finding is consistent with the idea that recycling should be completed before endosomes mature and fuse with the lysosome (Huotari and Helenius, 2011; Poteryaev et al., 2010).

A third possible scenario is that the permeases prefer ergosterol-rich domains for export to the plasma membrane. In the absence of ergosterol, cargo would be transported in a Glo3-dependent alternative route. However shutting down both pathways would cause the accumulation of the permeases in internal compartments.

The glucose transporter Hxt2 is degraded in the vacuole after endocytosis. Importantly, we did not observe an accumulation of Hxt2 in endosomes Δ*glo3*Δ*erg3* cells, under conditions under which Tat2, Gap1 and Snc1 were trapped internally. These data suggest that, similar to mammalian cells, different types of endosomes also exist in yeast, which would be dealing with different types of cargoes. Using correlative electron microscopy in the future might shed some light on the different types of endosomes.

## MATERIALS AND METHODS

### Yeast methods, strains and growth assays.

Standard yeast genetic techniques and media were used (Sherman, 1991). All strains, unless otherwise indicated, were grown at 30°C. HC (Hartwell's complete) medium selective for the plasmid was used to grow transformants. For experiments with proline as the sole nitrogen source, cells were grown first in HC media to OD_600_=0.5–0.7, washed twice in HC with 2 g/l of proline and incubated in the same medium at 30°C. Yeast strains used in this study are listed in supplementary material Table S1. The *glo3*::HIS3 deletion has been described previously (Poon et al., 1999). Chromosomal tagging and deletions were performed as described before (Gueldener et al., 2002). For co-staining of GFP-tagged proteins and the ER, the cells were transformed with the plasmid p424GPD-Sec63-RFP (kindly provided by S. Michaelis). For co-staining with Golgi apparatus, cells carrying a chromosomal 3×Cherry C-terminal insertion into Anp1 locus were used. For drop tests, cells were grown in liquid selective medium or YPD overnight, adjusted to OD_600_=0.1, and 10-fold serial dilutions were dropped onto agar plates. The plates were incubated at indicated temperatures for appropriate duration.

### Plasmids

All plasmids used in this study are listed in supplementary material Table S2. For expression of GFP-tagged Glo3, *GLO3* was amplified by PCR using yeast genomic DNA as template and cloned into pGFP33 (kindly provided by M. Hall) using *Xma*I and *Pst*I restriction sites. For yeast two-hybrid experiments, *GLO3* was amplified by PCR, digested with *Bam*HI and *Nco*I and cloned into the bait plasmid pEG202 (kindly provided by E. Schiebel). *PUB1*, *ARF1* and *ERG3* were PCR amplified, digested with *Eco*RI and *Xho*I and cloned into the prey plasmid pJG4-5 (kindly provided by E. Schiebel). For over-expression of *GLO3*, FLAG-tagged *GLO3* was amplified from pcDNA3.1-Glo3FLAG, digested with *Bam*HI and *Not*I and cloned into p424GPD (Euroscarf). For overexpression of *TAT2-GFP*, the gene was amplified from pKU76-Tat2-GFP, digested with *Eco*RI and *Sal*I and ligated into pRS426GPD. For *PMA1-GFP* overexpression, the insert was PCR-amplified, digested with *Sac*I and *Pst*I and cloned into pGFP195. For EM analysis, untagged Pma1 was produced by introducing a stop codon between *PMA1* and *GFP* in pGFP195-Pma1-GFP by using QuickChange Site-Directed Mutagenesis kit (Agilent Genomics). Sec63-RFP was amplified from pSM1959, cut with *Bam*HI and *Sal*I and cloned into p424GPD. The truncated *GLO3* versions were subcloned into p424GPD from the plasmids described previously (Schindler et al., 2009). The *GCS1* gene was amplified from genomic DNA and cloned into *Eco*RV-restricted p424GPD.

### HBH-purification

The HBH purification was carried out as previously described (Tagwerker et al., 2006) with modifications. Briefly, cells that expressed Glo3-HBH were grown to OD_600_=0.8–1.2 at 30°C. Cells were fixed by the addition of 1% formaldehyde for 2 min with gentle agitation at RT. The formaldehyde was quenched for 5 min with 1.25 mM glycine. Cells were harvested (4,700 ***g*** for 3 min at 4°C), washed in 50 ml ice-cold H_2_O, spun (3,000 ***g*** for 5 min at 4°C), flash-frozen in liquid nitrogen and stored at -80°C. The tandem affinity purification was performed as described (Ritz et al., 2014). The eluted proteins were subjected to endoproteinase LysC (ELC) cleavage, desalted and trypsin-digested as described (Weidner et al., 2014). The peptides were analyzed using LC-MS/MS.

### Yeast two hybrid and β*-*galactosidase assays

For yeast two-hybrid assay, Glo3 fused to LexA was used as bait (in pEG202) while the target proteins were fused to B42 protein (in pJG4-5). The interaction between each pair of proteins was measured by β-galactosidase assay, performed as described previously (Guarente, 1983). Activities were calculated as Miller units. Experiments were performed in triplicates.

### Analysis of chemical fragmentation and phosphorylation of Sch9

Cells expressing Sch9-3HA were collected at indicated time points following exposure to rapamycin and processed as described previously (Stracka et al., 2014). Cleavage of Sch9 by 2-nitro-5-thiocyanatobenzoic acid (NTCB) was carried out as described before (Urban et al., 2007). The products of the reaction were further analyzed by SDS PAGE and immunoblotting using anti-HA antibody (a kind gift of M. Hall).

### FM4-64 staining

Staining with the lypophilic dye FM4-64 was performed by incubating cells with 1× FM4-64 for 10 min at 30°C. Cells were harvested and incubated for 10 min in media without FM4-64 at 30°C. Cells were sedimented, mounted and analyzed immediately.

### Microscopy

Cells were grown to OD_600_=0.5–0.7 in YPD or HC medium supplemented with 50 mg/l adenine, harvested, and mounted. Images were acquired with an Axiocam mounted on a Zeiss Axioplan 2 fluorescence microscope. For electron microscopy, cells over-expressing Pma1 or Pma1-GFP were grown to OD_600_=0.5 at 30°C. Cells were fixed and treated for electron microscopy as described previously (Poon et al., 2001; Prescianotto-Baschong and Riezman, 2002). Image processing was performed using Image J and Adobe Photoshop CS3 (San Jose, CA, USA).

## Supplementary Material

Supplementary Material
