# Supplementary material for: The ArfGAP2/3 Glo3 and ergosterol collaborate in transport of a subset of cargoes
Source: Biol Open. 2015 May 11;4(7):792–802. doi: 10.1242/bio.011528 (PMC4571087; doi:10.1242/bio.011528)
Supplement: Supplementary Material [file supp_4_7_792__index.html]

The ArfGAP2/3 Glo3 and ergosterol collaborate in transport of a subset of cargoes — The ArfGAP2/3 Glo3 and ergosterol collaborate in transport of a subset of cargoes — Supplementary Material 

# The ArfGAP2/3 Glo3 and ergosterol collaborate in transport of a subset of cargoes

## BIO011528 Supplementary Material

- Supplementary Material
