# Supplementary material for: The ArfGAP2/3 Glo3 and ergosterol collaborate in transport of a subset of cargoes
Source: Biol Open. 2015 May 11;4(7):792–802. doi: 10.1242/bio.011528 (PMC4571087; doi:10.1242/bio.011528)
Supplement: Supplementary Material [file supp_bio.011528_BIO011528supp.pdf]

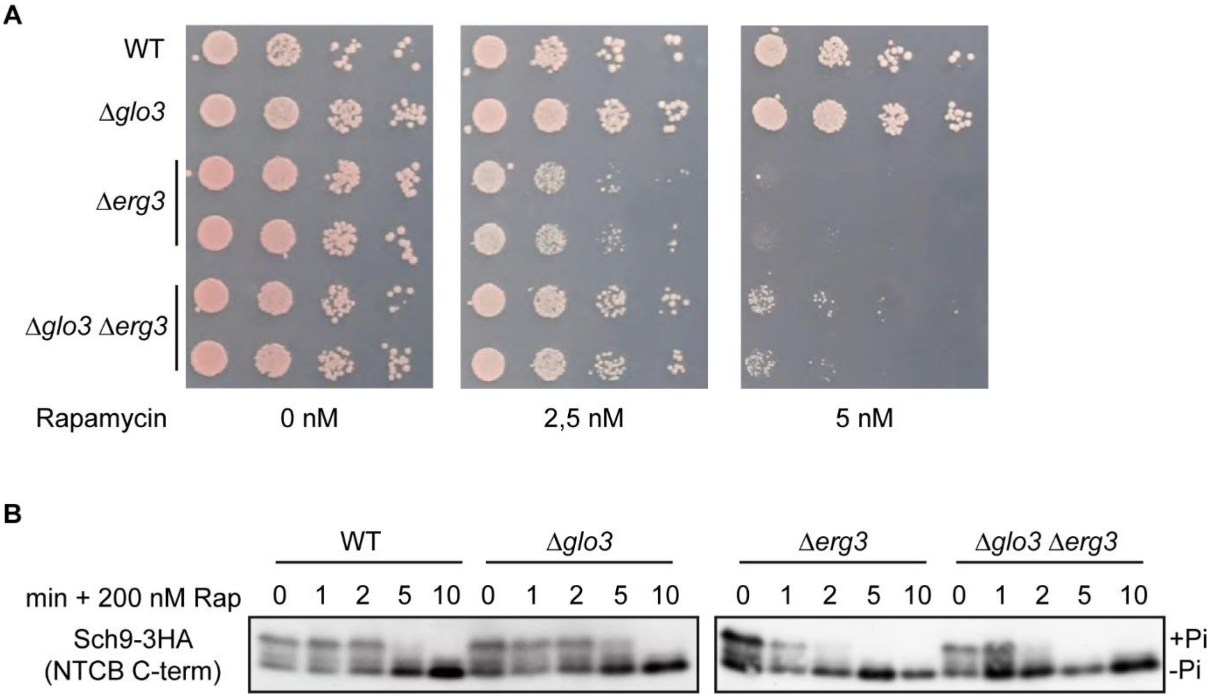

Supplementary material Fig. S1. Loss of Glo3 partially rescues the Δ*erg3* rapamycin sensitivity. (A) Δ*glo3*Δ*erg3* cells are less rapamycin-sensitive than Δ*erg3* cells. Drop assay of indicated strains on plates containing different concentrations of rapamycin. (B) Deletion of *GLO3* does not rescue the faster Sch9 dephosphorylation in Δ*erg3* cells upon rapamycin treatment. Immunoblot of Sch9-3HA C-terminal NTCB cleaved fragment after various times after rapamycin treatment. Sch9 is more rapidly dephosphorylated in Δ*erg3* cells when compared to wild type. This kinetics is not drastically altered in the Δ*glo3*Δ*erg3* double mutant.

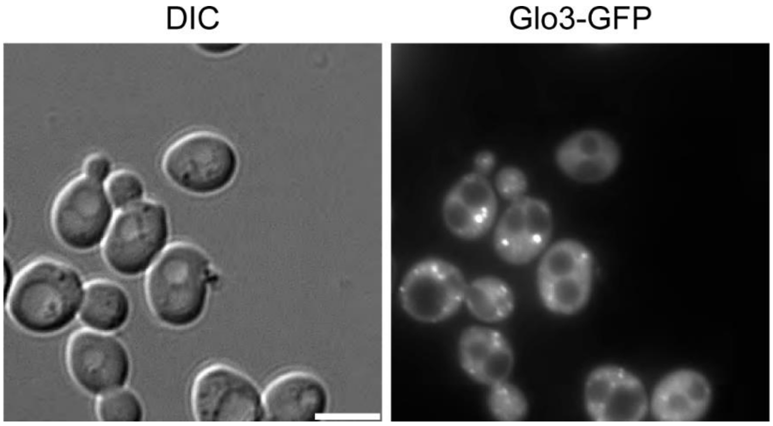

Supplementary material Fig. S2. Glo3 exists in a soluble and membrane associated state. Live cell imaging of cells expressing Glo3-GFP from the endogenous locus. Scale bar represents 5 µm.

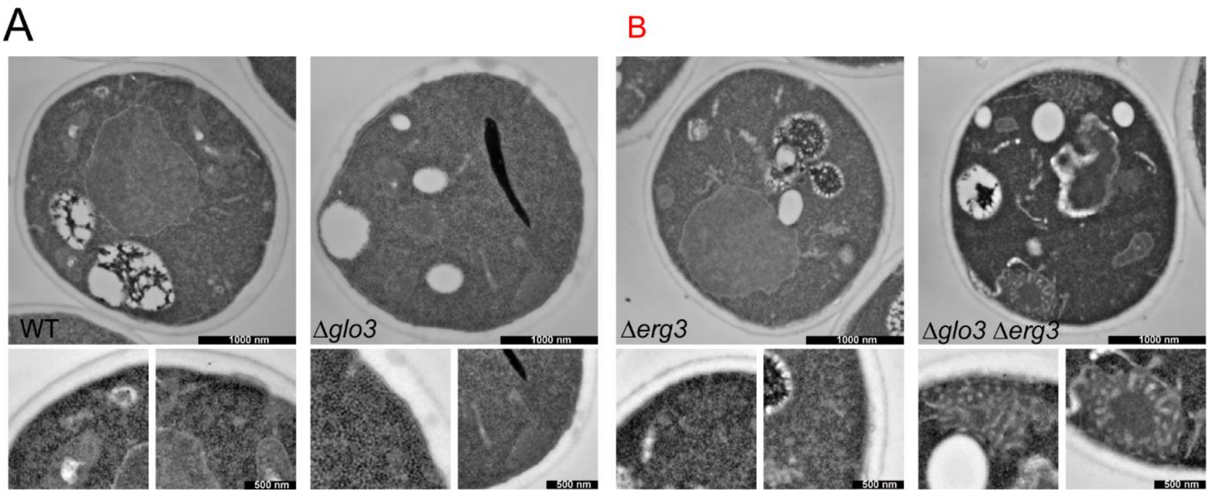

Supplementary material Fig. S3. Pma1 ER aggregation is not due to the GFP tag. Electron microscopy analysis of strains expressing Pma1 without a tag. The scale bar in the low magnification is 1 µm and for the enlargements 500 nm.

Table S1 Strains used in this study

Designation	Genotype	Source
RSY367	*MAT***a** *leu2-3,112 ura3-1 his3-11,15 trp1-1 ade2-1*	Randy Schekman
YAS2734	*MAT***a** *leu2-3,112 ura3-1 his3-11,15 trp1-1 ade2-1 GLO3::GLO3-HBH-TRP1*	This study
PPY51	*MAT***a** *leu2-3,112 ura3-1 his3-11,15 trp1-1 ade2-1 glo3::HIS3*	Pak Phi Poon
YAS3295	*MAT***a** *leu2-3,112 ura3-1 his3-11,15 trp1-1 ade2-1 erg3::LEU2*	This study
YAS3317	*MAT***a** *leu2-3,112 ura3-1 his3-11,15 trp1-1 ade2-1 glo3::HIS3 erg3::LEU2*	This study
YAS3103	*MAT***a** *leu2-3,112 ura3-1 his3-11,15 trp1-1 ade2-1 HXT2::HXT2-GFP-TRP1*	This study
YAS3104	*MAT***a** *leu2-3,112 ura3-1 his3-11,15 trp1-1 ade2-1 glo3::HIS3 HXT2::HXT2-GFP-TRP1*	This study
YAS3133	*MAT***a** *leu2-3,112 ura3-1 his3-11,15 trp1-1 ade2-1 erg3::LEU2 HXT2::HXT2-GFP-TRP1*	This study
YAS3105	*MAT***a** *leu2-3,112 ura3-1 his3-11,15 trp1-1 ade2-1 glo3::HIS3 erg3::LEU2 HXT2::HXT2-GFP-TRP1*	This study
YAS2969	*MAT***a** *leu2-3,112 ura3-1 his3-11,15 trp1-1 ade2-1 GLO3::GLO3-GFP-KanMX4*	This study

Table S2 Plasmids used in this study

Plasmid	Marker / Promoter	Source
pJG4-5-*ARF1*	TRP1, amp^R / GAL	This study
pEG202-*GLO3*	HIS, amp^R / ADH1	This study
pJG4-5-*PUB1*	TRP, amp^R / GAL	This study
pJG4-5-*ERG3*	TRP, amp^R / GAL	This study
pRS426*GPD*-Tat2-GFP	URA3, amp^R / GPD	This study
pRS416-Sch9-3HA	URA3, amp^R	Micheal N Hall
p426TEF-*GAP1-GFP*	URA3, amp^R / TEF	This study
pRS413	HIS3, amp^R	Micheal N Hall
YCplac22	TRP1, amp^R	Micheal N Hall
YCplacIII	LEU2, amp^R	Micheal N Hall
pRS315-Chs3-GFP	LEU2, amp^R	Cesar Roncero
pGAL39ΔBgIII-*CPY*-GFP	URA3, amp^R / GAL	Oliver Deloche
pGFP195-*PMA1*-GFP	URA3, amp^R	This study
pGFP195-*PMA1*	URA3, amp^R	This study
p424GPD-*SEC63*-RFP	TRP1, amp^R / GPD	This study
p416-*SNC1*-GFP	TRP1, amp^R	Nava Segev
p416-*SNC1PEM*-GFP	TRP1, amp^R	Nava Segev
p424GPD-*TRP1*	TRP1, amp^R / GPD	Euroscarf
p424GPD-*GLO3-TRP1*	TRP1, amp^R / GPD	This study
p424GPD- $glo3^{1\text{-}214}$-*TRP1*	TRP1, amp^R / GPD	This study
p424GPD-$glo3^{214\text{-}375}$-*TRP1*	TRP1, amp^R / GPD	This study
p424GPD- $glo3^{R59K}$ -*TRP1*	TRP1, amp^R / GPD	This study
p424GPD-*GCS1-TRP1*	TRP1, amp^R / GPD	This study
